# Monitoring progress in reducing maternal mortality using verbal autopsy methods in vital registration systems: what can we conclude about specific causes of maternal death?

**DOI:** 10.1186/s12916-019-1343-4

**Published:** 2019-06-03

**Authors:** Ian D. Riley, Riley H. Hazard, Rohina Joshi, Hafizur Rahman Chowdhury, Alan D. Lopez

**Affiliations:** 1 School of Population and Global Health, University of Melbourne, Parkville, VIC Australia; 2 The George Institute for Global Health, University of New South Wales, Sydney, Australia

**Keywords:** Verbal autopsy, Maternal, Civil registration and vital statistics, Sustainable development goals

## Abstract

Reducing maternal mortality is a key focus of development strategies and one of the indicators used to measure progress towards achieving the Sustainable Development Goals. In the absence of medical certification of the cause of deaths that occur in the community, verbal autopsy (VA) methods are the only available means to assess levels and trends of maternal deaths that occur outside health facilities. The 2016 World Health Organization VA Instrument facilitates the identification of eight specific causes of maternal death, yet maternal deaths are often unsupervised, leading to sparse and generally poor symptom reporting to inform a reliable diagnosis using VAs. There is little research evidence to support the reliable identification of specific causes of maternal death in the context of routine VAs. We recommend that routine VAs are only used to capture the event of a maternal death and that more detailed follow-up interviews are used to identify the specific causes.

**Electronic supplementary material:**

The online version of this article (10.1186/s12916-019-1343-4) contains supplementary material, which is available to authorized users.

## Background

The World Health Organization recommends the use of verbal autopsy (VA) methods to measure maternal mortality in countries without adequate medical certification of the cause of death, in part to enable them to monitor progress towards the Sustainable Development Goal of reducing the global maternal mortality ratio to less than 70 per 100,000 live births [1, 2]. This recommendation implies that procedures for the collection of VAs are in place to ensure the completeness of reporting of maternal deaths and that sufficient information about the cause of each maternal death from these sources is available to guide policy responses.

VAs were developed in Demographic Surveillance Sites in the 1980s and 1990s to identify leading causes of death in populations where the coverage of medical certification was limited [3]. The method was subsequently used for the assignment of cause of death for specific diseases for research purposes [4]. Over the last 4 years, the Bloomberg Data for Health Initiative to strengthen civil registration and vital statistics (CRVS) systems in low- and middle-income countries has facilitated the routine application of VAs in vital statistics systems to generate cause of death data for hitherto underserved populations [5]. Currently, VA questionnaires have been shortened, with less emphasis on open-ended responses due to inter-interview variability [6]. VA interviewers are commonly recruited from health services, with the collection of VAs representing an additional task.

Our experience assembled under the Bloomberg Data for Health Initiative over the past 4 years is that VAs collected through CRVS are sufficient to identify the leading 10–20 causes of death in low- and middle-income countries, including maternal deaths [5]. However, we argue that routine application of VAs in CRVS systems is not sufficient to obtain the necessary detailed information about the specific causes of maternal death that the World Health Organization 2016 VA Instrument aims to identify (Box 1) [7]. While it would clearly be desirable to be able to generate such epidemiological specificity from routine application of VAs in a CRVS system, we do not believe that these data can be collected with sufficient accuracy to be useful for public policy. Rather, we suggest that, in order to reliably describe the distribution of maternal causes in a population using VAs, a two-step process is required, involving (1) the identification of a maternal death using VAs and (2) the collection of detailed data by dedicated interviewers in a follow-up enquiry involving family and community collaboration.

### VAs for maternal deaths

The accuracy of VAs depends on four interdependent factors, namely (1) the informant’s experience of the terminal illness and the observation and interpretation of key signs and symptoms, (2) the skills, training, and motivation of interviewers, (3) the nature of the VA instrument, and (4) the method of analysis of the interview results.

A study from the Matlab Demographic Surveillance System research site in Bangladesh compared levels and causes of maternal mortality derived from routine collection of data with that arising from two VA special investigations [8]. Routine reporting identified only 67% of all deaths occurring during pregnancy or within 42 days postpartum. Further, the two VA specific studies disagreed in the ascertainment of the causes of maternal deaths and yielded different cause of death distributions. Moreover, there is empirical evidence to suggest that the identification of the causes of maternal death using routine VAs is likely to be very difficult. According to the authors, “*The female interviewer for the* [second] *special investigations was not only conscious of the need to specifically scrutinize all female deaths for their association with pregnancy, she spent substantial amounts of time listening to the relatives and neighbours of the deceased so as to carefully reconstruct the complex circumstances leading to the death. It is uncertain whether such intense efforts can be incorporated in a routine surveillance system*” ([8], p. 664).

In the Population Health Metrics Research Consortium VA gold standard study including 466 maternal deaths [9], VA data were collected from within hospital catchment areas and not from Matlab Demographic Surveillance System sites. Additional file 1 shows endorsement rates (i.e., the respondent had answered ‘yes’ to a question) for key symptoms associated with five maternal mortality diagnoses, namely hypertensive disorder, hemorrhage, sepsis, anemia, and other causes of maternal death. The symptom list is not comprehensive, but the distribution of responses is a good indication of the difficulties encountered in analyzing the data provided by respondents. The failure of the VA diagnostic method used to distinguish between these five causes was a direct consequence of the way that families had responded to the questionnaire. This is likely to be a common problem [10].

We could find only two published studies that claim to have established the validity for VAs in discriminating among maternal causes, both of which assume the deployment of specially trained interviewers [3, 11]. The first used hospital diagnoses as gold standard but validated the diagnostic algorithm by physician opinion [3]. We re-analyzed the data using statistical methods (Additional file 2) and found that, while there appeared to be reasonable agreement (mean chance-corrected concordance (individual level diagnostic) accuracy, 0.42 [12]; cause-specific mortality fraction (i.e., disease group) accuracy, 0.70 [13]) between the VA-assigned causes and the hospital diagnoses, there was substantial uncertainty due to a small sample size of only 80 total maternal deaths, with less than 18 for each specific maternal cause.

The second study validated a probabilistic model based on 80 ‘indicators’ for 21 causes of death in 258 women of reproductive age (15–49 years), where 77 deaths were pregnancy-related and 39 of these were due to direct maternal causes [11]. The authors provided no breakdown of indirect causes and showed direct causes diagrammatically but not tabulated, including only three specific causes (sepsis, obstructed labor, hemorrhage). The study’s strength was its analysis of community deaths, whereas it was limited by the disadvantages of convergent validation. Further, the study was too small to provide compelling evidence of the ability of the model to discriminate among direct and indirect maternal causes.

## Conclusions

Any death, and particularly a maternal death, is an emotional event for the family and relatives, but reporting and correctly diagnosing it is an important first step in providing the necessary health intelligence to guide health system responses. Our field experience regarding the ability of a VA interview and automated diagnostic algorithm to reliably distinguish between specific causes of maternal death, along with our interpretation of the very limited evidence, suggests that countries should be very cautious when assuming that VA methods designed for routine application are, inherently, sufficiently reliable to do so. All risk factors being equal, maternal mortality is highest for unsupervised deliveries [14]. Home deliveries often take place under adverse conditions and, when a woman is dying, panic may ensue. In such circumstances, it is unlikely that a (female) respondent to a VA interview will accurately recall, or even identify, some of the key symptoms required to adequately discriminate among maternal causes [15]. This would be even less likely if the VA respondent was a male who may or may not have been present when the events unfolded. Rather, accurate information about the causes of maternal death requires the development of a cadre of specially trained interviewers, the selection of one or more well-informed respondents, and careful, sympathetic questioning. Using VA to identify the causes of maternal deaths will be strengthened by the addition of separate semi-structured interviews focusing not only on the family, but also on health workers familiar with the details of the pregnancy. This is not current practice in countries trialing the routine application of VA in CRVS systems. Increasingly complex questionnaires and computer algorithms based on physician opinion will not solve the problem. Rather, we strongly recommend a two-step process of identifying deaths during routine CRVS followed by a detailed maternal mortality inquiry/survey. This paired, sequential approach to identifying the distribution of specific causes of maternal deaths will likely provide much more reliable evidence to better inform policy responses in countries as they strive to achieve the Sustainable Development Goal of reducing maternal mortality than data collected through VAs alone.

Box 1 Maternal causes of death included in the World Health Organization 2016 Verbal Autopsy Instrument Ectopic pregnancy

Abortion-related death

Pregnancy-induced hypertension

Obstetric hemorrhage

Obstructed labor

Pregnancy-related sepsis

Anemia of pregnancy

Ruptured uterus

Other and unspecified maternal cause

## Additional files

Additional file 1: Population Health Metrics Research Consortium (PHMRC) proportion of deaths positively endorsed (i.e., answered ‘yes’) for key symptoms for maternal death by cause (XLSX 10 kb)

Additional file 2: Specific maternal cause validation metrics. Performance indicators for a validation study of causes of maternal deaths in three African countries. (DOCX 13 kb)

## Abbreviations

CRVS: civil registration and vital statistics
VA: verbal autopsy
