# Supplementary material for: Monitoring progress in reducing maternal mortality using verbal autopsy methods in vital registration systems: what can we conclude about specific causes of maternal death?
Source: BMC Med. 2019 Jun 3;17:104. doi: 10.1186/s12916-019-1343-4 (PMC6545734; doi:10.1186/s12916-019-1343-4)
Supplement: Supplementary file 2 — Specific maternal cause validation metrics. Performance indicators for a validation study of causes of maternal deaths in three African countries. (DOCX 13 kb) [file 12916_2019_1343_MOESM2_ESM.docx]

| **Metric** | **Estimate (95% CI)** |
| --- | --- |
| Mean CCC | 0.42 (0.41, 0.43) |
| Median CCC | 0.45, (0.45, 0.45) |
| CSMF Accuracy | 0.70 (0.69, 0.71) |
| CCCSMF Accuracy | 0.17 (0.15, 0.20) |

| **Cause** | **Median CCC (95% CI)** |
| --- | --- |
| Abortion | 0.49 (0.48,0.51) |
| Acute febrile | 0.32 (0.31,0.35) |
| Anemia/CCF | -0.1 (-0.1, -0.1) |
| Eclampsia | 0.1 (0.10,0.12) |
| Hemorrhage | 0.56 (0.56,0.58) |
| Hepatitis | 0.63 (0.62,0.64) |
| Non-maternal | 0.94 (0.94,0.95) |
| Obstructed labor | 0.5 (0.49,0.52) |
| Other maternal | 0.45 (0.44,0.45) |
| Sepsis | 0.26 (0.25,0.27) |
| TB/AIDS | 0.53 (0.51,0.56) |

Abbreviations: CCC (chance corrected concordance), CSMF (cause-specific mortality fraction), CCCSMF (chance-corrected cause specific mortality fraction), CCF (congestive cardiac failure)
